# Identification of tumour-related proteins as potential screening markers by proteome analysis—protein profiles of human saliva as a predictive and prognostic tool

**DOI:** 10.1186/1878-5085-5-20

**Published:** 2014-11-28

**Authors:** Kurt Krapfenbauer, Elisabeth Drucker, Dietmar Thurnher

**Affiliations:** Department of Otorhinolaryngology, Head and Neck Surgery, Medical University of Vienna, A-1090 Vienna, Austria; Department of Molecular Biotechnology, University of Applied Science Vienna, Helmut-Qualtinger-Gasse 2, A-1090 Vienna, Austria

**Keywords:** Oral squamous cell carcinoma, Saliva proteomics, Predictive medicine, Targeted prevention, Tailored therapy, Individual patient profiles, Disease monitoring, Patient self-management

## Abstract

The analysis of biomarkers in saliva as a clinical application offers an attractive, simple and rapid diagnostic tool for the short- and long-term monitoring of pathological disorders and drug therapy. The collection of saliva, either in the pure or in its fractionated form, is a relatively easy and non-invasive procedure that is not harmful to the patients and has no complications at all. However, the fluid collection must be clearly defined due to variations in saliva composition, flow rate and day-to-day variability. In order to minimise possible variations, saliva from five patients without squamous cell carcinoma (SCC) pathology and five with suspicion of oral squamous carcinoma (OSCC) were collected and matched at different days and analysed by two-dimensional polyacrylamide gel electrophoresis (2DE-PAGE). Approximately 800 spots were identified, corresponding to 151 different gene products. The list of identified proteins includes a large number of structural proteins like keratins, keratin subunits, enzymes and enzyme inhibitors, cytokines, immunoglobulins as well as amylase and other salivary specific glycoproteins. The majority of proteins that are localised in oral epithelia cells were found as unsolved debris in saliva. One of the identified proteins was significantly overexpressed in OSCC and was selected for further validation by Western blot analysis.

## Overview

Head and neck cancers, a group of malignant neoplasias, are one of the most common forms of cancer worldwide [1] and most frequently occur as squamous cell carcinoma (SCC) of the mouth, pharynx or larynx. Oral squamous cell carcinomas (OSCC) have a poor prognosis with a 5-year survival rate of 30%–50%, which has not changed fundamentally over the last decades [2]. This is partly due to the fact that OSCC usually develops from asymptomatic lesions and is often diagnosed or treated only when it reaches an advanced state. The identification of markers to discriminate tumour from healthy cells already at the earliest stages of malignancy is of critical importance for clinical diagnosis, as is a reliable differentiation of the tumour stage.

In the past, gene and protein expression profiles from OSCC tumours have been reported in various studies using either tumour-derived cell lines [3] or tumour tissue [4]. Differential expression of genes or proteins in tumour vs. healthy tissue has revealed abundant biomarker candidates. Some of the genes or proteins have been reported repeatedly, like metalloproteinases, urokinase or laminins [5]. Due to differences in methodology and sample size, these findings are difficult to interpret and no consensus has been reached as to what markers are suitable to identify early malignant lesions. Therefore, oncologists still rely on the classical diagnosis [6]. Furthermore, these studies analysed tumour markers in tissue biopsies. The acquired information, though important for the staging of the tumour and for understanding mechanisms and pathways involved in carcinogenesis, was only of limited use for the discovery of screening biomarkers, as brush biopsy, the only non-invasive method to obtain tumour tissue, is a time-consuming method requiring highly trained personnel. Ultimately, diagnosis would be much improved if reliable tumour markers for OSCC could be identified in the periphery and sensitive and reliable routine testing could be performed either in blood or saliva samples. Consequently, circulatory tumour markers for OSCC have become a focus of research and have been subject to several studies [7–16]. However, the sensitivity and specificity of these markers are still rather poor. While some researchers found a combination of four biomarkers (CEA, SCC, immune-suppressive acidic protein, Cyfra) to be rather sensitive (81%) and specific (77.8%) [17] and even the first three markers alone were still rather sensitive (69%) and even more specific (90.3%) [17, 18], others using a different combination of markers (SCC, CEA, C19-9, CA125) found a significant correlation only for SCC [15, 16]. However, they pointed out that SCC sensitivity was rather low (15%–40%), although its specificity was quite high (70%–90%). Other studies reported even lower sensitivity and specificity for these markers. Thus, even a panel of peripheral biomarkers for OSCC does not provide sufficient sensitivity and specificity to serve as a diagnostic test yet.

Changes in DNA associated with cancer frequently translate into alterations in mRNA expression patterns. Since these changes occur early in cancerogenesis they might facilitate the detection of malignant events before they can be detected by other methods. Furthermore, contrary to proteins, nucleic acids can easily be amplified and therefore detected more easily. Consequently, various studies have analysed mRNA expression signature for their applicability as biomarkers of OSCC in tissue biopsies either by quantitative RT-PCR or by microarray experiments [19]. Microarray techniques are powerful means of generating a lot of analytical data in the frame of a single experiment. This analytical information is used to understand the nuance of the genome of a biological system and is the basis of comparison of two or more samples in many cases. Yet the technical difficulty and high cost of data production, associated with highly time-consuming data analysis, has contributed to a position where poor experimental design is common. Many experiments that use microarrays have a low number of analytical and/or biological replicates, and users of differential displays often assume that multiple estimates of differences generated by a single microarray experiment provide a substitute for experimental replicas. The reproducibility of the used microarray techniques, as assayed by regression analysis, co-efficient of variation or other variance estimation techniques [20], is typically not reported. Power analysis, which can be used to infer the number of samples required to discover a statistically significant result [20–22], are rarely undertaken. Weak experimental design, particularly in a field where technical challenges remain in the production of high-quality data, can make it difficult or impossible to determine if differences reported between two or more samples are likely to reflect variations in a biological system or are solely analytically derived.

Most studies published so far have analysed OSCC proteomics or genomics in tissue biopsies. Though the information gained from such studies is important for understanding the mechanisms of carcinogenesis and can lead to the identification of biomarkers or therapeutic targets, biopsy samples are not suitable for screening purposes. For early diagnosis and screening of risk populations, circulatory markers that can predict the development of malignancies at an early or even precancerous stage would be invaluable. Therefore, biomarkers in blood and, especially in the case of OSCC, saliva are now a focus of research. Though not all proteins identified in a tumour will be present in the periphery even those that are might be at a level below the current detection limits. Therefore, it is important to integrate the knowledge of tumour and saliva proteomics. Apart from proteins, blood and other body fluids contain both DNA and RNA which have been used as cancer markers and can be used to screen for new and specific biomarkers. A few studies so far have analysed the gene expression profile of OSCC in saliva samples [23].

The genetic aberrations in cancer cells lead to altered gene expression patterns, which can be identified long before the resulting cancer phenotypes are manifested. Changes that arise exclusively or preferentially in cancer, compared with normal tissue of the same origin, can be used as molecular biomarkers [24]. Accurately identified, biomarkers may provide new avenues and constitute major targets for early detection of cancer and cancer risk assessment. A variety of nucleic acid-based biomarkers have been demonstrated as novel and powerful tools for the detection of cancers [25–27]. However, most of these markers have been identified either in cancer cell lines or in biopsy specimens from late invasive and metastatic cancers. The ability to detect cancer in its earliest stages using/utilising biomarkers is still limited. Moreover, the invasive nature of a biopsy makes it unsuitable for cancer screening in high-risk populations. This suggests a crucial need for the development of new diagnostic tools that improve early detection. The identification of molecular markers in body fluids that predict the development of cancer already in earliest or precancerous stages would constitute such a tool. It has been shown that an identical mutation as present in the primary tumour can be identified in the body fluids tested from affected patients [28]. Cancer-related nucleic acids in blood, urine, and cerebrospinal fluid have been used as biomarkers for cancer diagnosis [29–31]. More recently, mRNA biomarkers in serum or plasma have been targets for reverse transcription PCR (RT-PCR)-based detection strategies in patients with cancers [32, 33]. Parallel to the increasing number of identified biomarkers in body fluids is the growing availability of more powerful and cost-efficient technologies that enable mass screening for genetic alterations. Recent discovery of the existence of a large panel of human mRNA in saliva by microarray technology [23] suggests a novel clinical approach, so-called salivary transcriptome diagnostics, for applications in disease diagnostics as well as for normal health surveillance. It is a high-throughput, robust and reproducible approach to harness RNA signatures from saliva. Moreover, using saliva as a diagnostic fluid meets the demands for inexpensive, non-invasive and accessible diagnostic methodology [34]. In the present study, the hypothesis that protein expression patterns can be identified in the saliva of cancer patients and that the differentially expressed transcripts can serve as biomarkers for cancer detection was tested. The proof-of-principle disease in this study is OSCC. The rationale is that oral cancer cells are immersed in the salivary milieu and genetic heterogeneity has been detected in the saliva from patients with OSCC [35, 36].

## Methods

### Materials

Immobilised pH gradient (IPG) strips and IPG buffers were purchased from Bio-Rad Laboratories (Hercules, CA, USA). Acrylamide/piperazine-di-acrylamide (PDA) solution (37.5:1, *w*/*v*) was purchased from Biosolve Ltd. (Valkenswaard, The Netherlands), and the other reagents for the polyacrylamide gel preparation were acquired from Bio-Rad Laboratories. CHAPS was obtained from Roche Diagnostics (Mannheim, Germany), urea from AppliChem (Darmstadt, Germany), thiourea from Fluka (Buchs, Switzerland), 1,4-dithioerythritol (DTE) and EDTA from Merck (Darmstadt, Germany) and tributylphosphine (TBP) from Pierce Biotechnology (Rockford, IL, USA). All reagents were kept at 4°C.

### Sample preparation

Saliva from three male and two female patients with OSCC as well as from ten healthy control persons were obtained from the General Hospital Vienna (Medical University of Vienna). The patients were between 42.3 and 64.2 years old. The stage of cancer was defined by the extent of the lesion and was determined by physical examination, radiological studies and pathological analysis and showed the presence of 66% ± 34% tumour cells in each tumour centre. Tumour species from two OSCC patients showed a stage of T3 (1 N0, 6 N2) M0, and from three patients, a stage of T4 (1 N0, 2 N2) M0. No tumour cells were detected in the surrounding mucosal tissue. The study was approved by the ethical committee of the General Hospital Vienna.

### Protocol for saliva collection and storage for using pre-coated sampling tubes

#### Sample collection

Human saliva samples were collected from control and OSCC donors, who spitted into a pre-coated tube after intensive tooth brushing and rinsing the mouth with water.

Tubes were coated with EDTA, serine, cysteine and metalloproteases as well as with calpains and a mixture of specific phosphor inhibitors. Due to the optimised composition of the pre-coated tubes and based on previous experiments, the mixture showed excellent inhibition effects and was therefore very well suited for the protection of saliva proteins directly isolated from the obtained human samples. It is important to mention that the inhibitor cocktail contained both irreversible and reversible protease inhibitors. The protease inhibitors present in the pre-coated tubes did not form irreversible complexes with the SH groups of proteins. Consequently, the addition of a protease inhibitor cocktail to all further stock buffers and solutions normally protected with protease and phosphotase inhibitors - not only during the initial saliva collection steps - is recommended in the present study. Eppendorf tubes were first coated with EDTA (0.37 mg/tube ≡ 1 mM), and then with aprotinin, Bestatin, calpain inhibitor I, calpain inhibitor II, chymostatin, E-64, leupeptin, α_2_-macroglobulin, Pefabloc SC, pepstain, PMSF, TLCK-HCL and trypsin inhibitors (each 1 μmol/ml). Additionally to the normally protease inhibitor cocktail, 1 μmol NaF and 10 μmol NaVO_3_ were used as specific phosphatase inhibitors in the tubes. The inhibitory power of the tube was checked with fresh blood samples in combination with proteases and protease mixtures. In this experiment, a drastically higher concentration of protease compared to the concentration usually present in normal blood samples was used. The inhibitory activity of these tubes was further tested with a concentrated blood extract and a concentrated protenase solution. The activity was determined using the Roche Applied Science Universal Protease Substrate. Thereby, the proteolytic activities were typically inhibited by 95% after 1 h at RT and by 90% after 24 h at 4°C. Similar effects can be assumed for saliva samples.

One tube proved to be sufficient for the inhibition of the proteolytic activity of 10 ml saliva. If very high proteolytic activity was present, less than 5 ml saliva/tube were collected.

After sample collection, the tubes were gently shacked (not vortexed!!) for approximately 1 min and snap-frozen in liquid nitrogen.

Saliva samples were kept at −80°C, and the freezing chain was maintained until analysis. To separate the supernatant from the cell debris, the samples (*ca.* 1.5 mL) were centrifuged at 3.000 × *g* for 15 min at 4°C and the cell pellets were washed three times with 20 mM HEPES, pH 7.5, containing 320 mM sucrose, 1 mM EDTA, 5 mM DTE, protease inhibitor cocktail (Roche Diagnostics, one tablet per 50 ml solution), 1 mM PMSF, 0.2 mM Na_3_VO_3_ and 1 mM NaF (homogenisation buffer). Suspensions were transferred in 40 mM Tris, containing 7 M urea, 2 M thiourea, 4% CHAPS, 10 mM 1,4-dithioerythriol, 1 mM EDTA, homogenised using a glass-Teflon potter (20 strokes at 200 rpm, 4°C) and centrifuged at 100,000 × *g* for 30 min to sediment not dissolved material [4].

#### Two-dimensional electrophoresis

Samples were desalted using membrane filter tubes (Merck Millipore, Amicon Ultra-0.5 mL Centrifugal Filters). The protein content in the supernatant was determined by the Coomassie blue method and revealed concentrations between 8–12 mg/ml. One or two mg of total protein was applied on immobilised pH 3–10 non-linear gradient strips, at both the basic and acidic ends. Focusing started at 200 V after which the voltage was gradually increased to 5,000 V at 3 V/min and continued at 5,000 V for 24 h. The second dimensional separation was performed in 12% polyacrylamide gels. After protein fixation with 50% (*v*/*v*) methanol containing 5% (*v*/*v*) phosphoric acid for 12 h, the gels were stained with colloidal Coomassie blue (Novex, San Diego, CA, USA) for further 24 h. The gels were destained with H_2_O and scanned in an Agfa densitometer (Agfa-Gevaert N.V., Mortsel, Belgium). The images were processed using Photoshop (Adobe, San Jose, CA, USA) and PowerPoint (Microsoft, Redmond, WA, USA) software. Protein spots were quantified using the Image Master 2D Elite software (BioRad Laboratories, Hercules, CA, USA). The percentage of the volume of the spots representing a certain protein was determined in comparison with the total proteins present in the 2-D gel.

### Matrix-assisted laser desorption ionisation time-of-flight mass spectrometry

Protein identification was performed by matrix-assisted laser desorption ionization time-of-flight mass spectrometry (MALDI-TOF-MS) as previously described [4, 5] but with minor modifications. Briefly, spots were excised, destained with 30% (*v*/*v*) acetonitrile in 0.1 M ammonium bicarbonate and dried in a Speedvac evaporator (Thermo Scientific, Waltham, MA, USA). The dried gel pieces were rehydrated with 5 μl of 5 mM ammonium bicarbonate (pH 8.8) containing 50 ng trypsin (Promega, Madison, WI, USA), centrifuged for 1 min and left at room temperature for about 12 h. After digestion, 5 μl of water were added, followed by 10 μl of 75% acetonitrile containing 0.3% trifluoroacetic acid 10 min later, centrifuged for 1 min and the content was vortexed for 2 min. Of the separated liquid, 1.5 μl was mixed with 1 μl of saturated alpha-cyano-4-hydrocinnamic acid in 50% acetonitrile and 0.1% TFA in water and applied to the sample target. The samples were analysed in a time-of-flight mass spectrometer (Ultraflex, Bruker Daltonics) equipped with a reflector and delayed extraction. An accelerating voltage of 20 kV was used. Calibration was internal to the samples. Des-Arg-1 bradykinin (Sigma-Aldrich, St. Louis, MO, USA) and ACTH (Sigma) were used as standard peptides. The peptide masses were matched with the theoretical peptide masses of all proteins from all species of the SWISS-Prot database. For protein search, monoisotopic masses were used and a mass tolerance of 0.0025% was allowed. The protein search was performed using an in-house developed software [28]. Proteins not identified in the MS mode were further characterised by MALDI LIFT-TOF/TOF MS experiments. Thereby, a relatively low voltage of 8 kV was initially applied for ion acceleration. Fragments generated from laser-induced dissociation were subsequently raised to a higher potential (19 kV) in the LIFT cell.

### Western blot analysis

Monoclonal antibody (anti-human Gal-7, BioVisions Cat.Nr. 5647–100) was diluted 1:5,000 in blocking buffer, applied to the membranes and incubated together for 1 h at 25°C on an orbital shaker. After primary incubation, the membranes were washed three times for 10 min in 0.05% Tween 20 in PBS and finally one time for 10 min in PBS only.

After blocking, subsequently, the specifically bound primary antibody was conjugated with a horseradish peroxidase-conjugated secondary antibody. For detection of the bound horseradish peroxidase-conjugated monoclonal antibody, the membrane was incubated with SuperSignal WestFemto Maximum Sensitivity Substrate. The working solution was prepared by mixing equal parts of the stable peroxide solution and the luminal/enhancer solution. The 0.125 ml/cm^2^ working solution of membrane was used. The working solution was stable for 24 h at room temperature; however, fresh preparation prior to incubation is strongly recommended.

## Results

**Figure 1:**
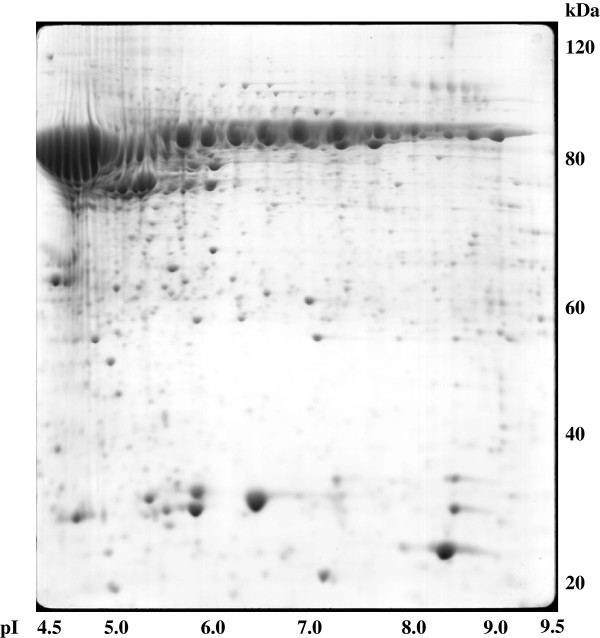
**Two-dimensional map of human saliva proteins.** Epithelial cell proteins from control patients were isolated from saliva by different sucrose centrifugation steps and separated on pH 3–10 non-linear IPG strip, followed by 12% SDS-polyacrylamide gel. The gel was stained with Coomassie blue. The spots were analysed by MALDI-MS, and the names of identified proteins are listed in Table 1.

**Figure 2:**
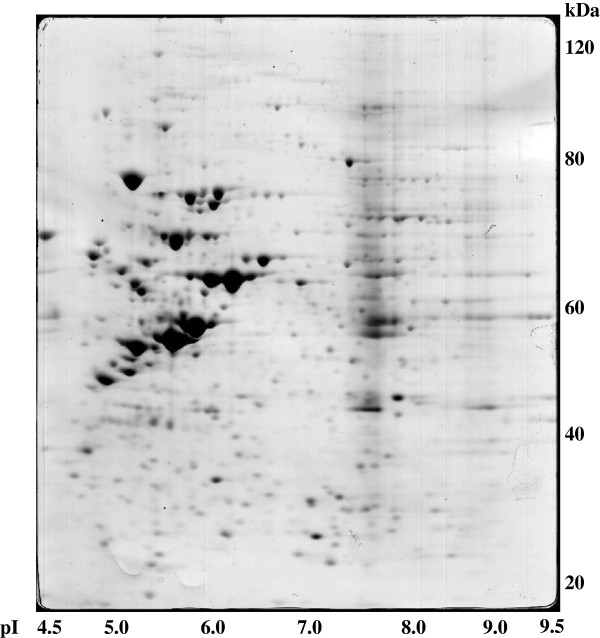
**Two-dimensional map of human saliva proteins.** Supernatant from saliva samples were isolated by centrifugation and separated on pH 3–10 non-linear IPG strip, followed by 12% SDS-polyacrylamide gel. The gel was stained with Coomassie blue. The spots were analysed by MALDI-MS, and the name of identified proteins are listed in Table 1.

The fractions enriched in supernatant and cell debris proteins from saliva samples were analysed by two-dimensional electrophoresis using broad pH range 3–10 IPG strips. The gels were stained with Coomassie blue, following the standard protocol already described in [4, 5] because this approach enables identification rates up to 90%. Furthermore, Coomassie blue is more suitable for protein quantification from gels since it shows a linear dynamic range in comparison with silver stain. Silver stain is more sensitive but exhibits a non-linear dynamic range and a tendency to stain differently based on the amino acid composition and post-translational modifications of a given protein. Up to 800 spots were detected on each gel (Figures 1 and 2). For the construction of a two-dimensional protein database for saliva, approximately 21,000 spots were excised from 2 × 10 2-D gels and the proteins were identified by MALDI-TOF-MS. Proteins that could not be identified in the MS mode were analysed in the TOF/TOF mode. Whereas the acquisition of a post-source decay (PSD) spectrum may take considerable time (approximately 20 min) when stepping down the reflector voltage, the product ions from laser-induced metastable decay can be recorded in a MALDI TOF/TOF instrument employing the LIFT technology. This enables the rapid (seconds) detection of all fragments without changing the reflector voltage, which, compared to conventional PSD, is particularly advantageous for the detection of low mass ions of low abundance. The identified proteins were the products of different genes. Further, the major differences in the protein level in the supernatant and cell debris fractions from cancer patients and healthy individuals were studied.

Saliva samples from five control persons and five patients with suspicion of oral squamous carcinoma were investigated. Approximately 800 spots/gel were identified, corresponding to 151 different gene products. The list of identified proteins (see Table 1) included a large number of structural proteins like keratins, keratin subunits, enzymes and enzyme inhibitors, cytokines, immunoglobulins as well as amylase and other salivary specific glycoproteins. The majority of the identified proteins had their origin in oral epithelia cells and were found as unsolved debris in saliva (see Figures 1 and 2). Twenty-five proteins seemed to be specific for SCC and were identified in the saliva of all patients with suspicion of OSCC, but not in healthy individuals, by two-dimensional polyacrylamide gel electrophoresis (2DE-PAGE) (see Table 2).

**Table 1:** **List 1: identified proteins in human saliva**

Loc.	Swiss-Prot.-Nr.	Mat.	pMism.	Protein name
B	CHR14-ALC2	5	pMism:7.51	Ig alpha-2 chain C region was used to identify this gene
B	CHR5-MY10	6	pMism:6.88	Myosin X was used to identify this gene
B	CHR20-CAC24982	5	pMism:8.84	tr:cac24982: sequence 26 from patent wo0100806 precursor was used to identify this gene
A	CHR1-CAC32430	7	pMism:10.65	tr:cac32430: sequence 17 from patent wo0105971 was used to identify this gene
A	CHR12-CAD48670	7	pMism:8.20	tr:cad48670: sequence 1 from patent wo0229058 was used to identify this gene
A	CHR11-Q14697	9	pMism:7.93	tr:q14697: glucosidase II precursor (kiaa0088 protein) was used to identify this gene
A	CHR12-Q8N1N4	6	pMism:7.53	tr:q8n1n4: hypothetical protein FLJ39100 was used to identify this gene
A	CHR1-Q8N613	9	pMism:9.63	tr:q8n613: chromosome 1 open reading frame 10 was used to identify this gene
A	CHRX-Q8NG12	9	pMism:12.95	tr:q8ng12: premature ovarian failure 1b protein was used to identify this gene
A	CHR14-Q96HE7	8	pMism:12.21	tr:q96he7: ERO1 (*S. cerevisiae*)-like was used to identify this gene
A	SW:143S_HUMAN	5	pMism:11.49	14-3-3 protein sigma (stratifin) (epithelial cell marker protein 1)
A	SW:143Z_HUMAN	6	pMism:9.24	14-3-3 protein zeta/delta (protein kinase C inhibitor protein 1)
A	SW:GR78_HUMAN	7	pMism:8.88	78 kDa glucose-regulated protein precursor (GRP 78)
A, B	SW:ACTS_HUMAN	5	pMism:8.20	Actin, alpha skeletal muscle (alpha-actin 1)
A, B	SW:ACTB_HUMAN	9	pMism:14.16	Actin, cytoplasmic 1 (beta-actin)
A	SW:ARP3_HUMAN	6	pMism:8.94	Actin-like protein 3 (actin-related protein 3) (actin-2)
A	SW:ADK_HUMAN	5	pMism:8.34	Adenosine kinase (EC 2.7.1.20) (ak)
A	SW:SAHH_HUMAN	6	pMism:8.76	Adenosylhomocysteinase (EC 3.3.1.1)
A	SW:ALDX_HUMAN	4	pMism:7.16	Alcohol dehydrogenase [NADP+] (EC 1.1.1.2)
A	SW:CRAB_HUMAN	4	pMism:10.61	Alpha crystallin B chain (alpha(B)-crystallin) (rosenthal fiber component)
A, B	SW:ENOA_HUMAN	9	pMism:13.48	Alpha enolase (EC 4.2.1.11) (2-phospho-d-glycerate hydro-lyase)
A, B	SW:ENOL_HUMAN	5	pMism:7.02	Alpha enolase, lung specific (EC 4.2.1.11)
B	SW:A2HS_BOVIN	6	pMism:9.32	Alpha-2-HS-glycoprotein precursor (Fetuin-a) (asialofetuin)
A	SW:AAC4_HUMAN	8	pMism:9.52	Alpha-actinin 4 (non-muscle alpha-actinin 4)
B	SW:AMYC_HUMAN	9	pMism:9.61	Alpha-amylase 2b precursor (EC 3.2.1.1)
A, B	SW:AMYS_HUMAN	9	pMism:13.68	Alpha-amylase, salivary precursor (EC 3.2.1.1)
A	SW:ACTZ_HUMAN	4	pMism:10.27	Alpha-centractin (centractin) (centrosome-associated actin homolog)
A	SW:ANX4_HUMAN	9	pMism:14.41	Annexin A4 (annexin IV) (Lipocortin IV) (Endonexin I)
A	SW:ANX8_HUMAN	1	pMism:15.43	Annexin A8 (annexin VIII) (vascular anticoagulant-beta)
A	SW:ANX1_HUMAN	9	pMism:17.87	Annexin I (lipocortin I) (calpactin II) (chromobindin 9) (p35)
A	SW:ANX2_HUMAN	9	pMism:16.80	Annexin II (lipocortin II) (calpactin I heavy chain)
A	SW:ANX3_HUMAN	8	pMism:12.81	Annexin III (lipocortin III) (placental anticoagulant protein III)
A	SW:ANX6_HUMAN	8	pMism:11.62	Annexin VI (lipocortin VI) (p68) (p70) (protein III) (chromobindin 20)
A, B	SW:AOP2_HUMAN	6	pMism:13.04	Antioxidant protein 2 (1-cys peroxiredoxin) (1-cys Prx)
B	SW:APA1_HUMAN	6	pMism:9.19	Apolipoprotein a-i precursor (Apo-AI)
A	SW:ATPA_HUMAN	4	pMism:7.38	ATP synthase alpha chain, mitochondrial precursor (EC 3.6.3.14)
A	SW:ATPB_HUMAN	7	pMism:7.00	ATP synthase beta chain, mitochondrial precursor (EC 3.6.3.14)
B	SW:ENOB_HUMAN	6	pMism:8.88	Beta enolase (EC 4.2.1.11) (2-phospho-D-glycerate hydro-lyase)
A	SW:PMGE_HUMAN	6	pMism:14.78	Bisphosphoglycerate mutase (EC 5.4.2.4)
A, B	SW:S108_HUMAN	4	pMism:9.18	Calgranulin A (migration inhibitory factor-related protein 8) (MRP 8)
A, B	SW:S109_HUMAN	6	pMism:11.89	Calgranulin B (migration inhibitory factor-related protein 14) (MRP 14)
A, B	SW:CAH6_HUMAN	8	pMism:17.32	Carbonic anhydrase VI precursor (EC 4.2.1.1) (carbonate dehydratase VI)
A	SW:DHCA_HUMAN	6	pMism:10.55	Carbonyl reductase [NADPH] 1 (EC 1.1.1.184) (NADPH-dependent carbonyl reductase 1)
B	SW:CATA_HUMAN	10	pMism:14.91	Catalase (EC 1.11.1.6)
A	SW:CLI1_HUMAN	8	pMism:13.04	Chloride intracellular channel protein 1 (nuclear chloride ion channel 27)
A	SW:CLI3_HUMAN	6	pMism:14.75	Chloride intracellular channel protein 3
A	SW:COF1_HUMAN	4	pMism:8.17	Cofilin, non-muscle isoform (18 kDa phosphoprotein) (p18)
B	SW:CO3_HUMAN	9	pMism:8.91	Complement C3 precursor [contains: C3a anaphylatoxin]
A, B	SW:CO1A_HUMAN	7	pMism:13.63	Coronin-like protein p57 (coronin 1A)
A	SW:CYTB_HUMAN	4	pMism:8.79	Cystatin B (liver thiol proteinase inhibitor) (CPI-B) (stefin B)
B	SW:CYTD_HUMAN	4	pMism:7.88	Cystatin D precursor
A, B	SW:CYTS_HUMAN	7	pMism:16.66	*Cystatin S precursor (salivary acidic protein-1) (cystatin SA-III)*
B	SW:CYTT_HUMAN	6	pMism:11.57	Cystatin SA precursor (cystatin S5)
B	SW:CYTN_HUMAN	5	pMism:8.38	Cystatin SN precursor (salivary cystatin SA-1) (cystain SA-I)
B	SW:CRS3_HUMAN	4	pMism:7.74	Cysteine-rich secretory protein-3 precursor (CRISP-3) (SGP28 protein)
B	SW:DSC2_HUMAN	8	pMism:10.10	Desmocollin 2A/2B precursor (desmosomal glycoprotein II and III)
A	SW:EML2_HUMAN	6	pMism:8.38	Echinoderm microtubule-associated protein-like 2 (EMAP-2)
A	SW:EF11_HUMAN	4	pMism:8.12	Elongation factor 1-alpha 1 (EF-1-alpha-1) (elongation factor 1 a-1)
A	SW:EFTS_NEIMA	6	pMism:9.75	Elongation factor ts (EF-Ts)
A	SW:ER29_HUMAN	5	pMism:9.47	Endoplasmic reticulum protein ERp29 precursor (ERp31)
A	SW:ECHM_HUMAN	6	pMism:9.15	Enoyl-CoA hydratase, mitochondrial precursor (EC 4.2.1.17)
A	SW:IF32_HUMAN	6	pMism:11.90	Eukaryotic translation initiation factor 3 subunit 2 (eIF-3 beta)
A, B	SW:CAZ1_HUMAN	6	pMism:14.51	F-actin capping protein alpha-1 subunit (capZ alpha-1)
A	SW:CAZ2_HUMAN	5	pMism:14.83	F-actin capping protein alpha-2 subunit (capZ alpha-2)
A	SW:CAPB_HUMAN	5	pMism:8.35	F-actin capping protein beta subunit (capZ beta)
A	SW:FABE_HUMAN	5	pMism:10.80	Fatty acid-binding protein, epidermal (E-FABP)
B	SW:FIBB_HUMAN	8	pMism:14.03	Fibrinogen beta chain precursor.
A	SW:ALFA_HUMAN	6	pMism:10.30	Fructose-bisphosphate aldolase a (EC 4.1.2.13) (muscle-type aldolase)
A	SW:LEG3_HUMAN	5	pMism:9.79	Galectin-3 (galactose-specific lectin 3) (MAC-2 antigen) (IgE-binding protein)
A	SW:LEG7_HUMAN	5	pMism:8.58	Galectin-7 (HKL-14) (PI7)
B	SW:GELS_HUMAN	4	pMism:7.50	Gelsolin precursor, plasma (actin-depolymerizing factor) (ADF)
A	SW:GLNA_HUMAN	4	pMism:8.41	Glutamine synthetase (EC 6.3.1.2) (glutamate-ammonia ligase)
A, B	SW:GTP_HUMAN	8	pMism:14.25	Glutathione S-transferase p (EC 2.5.1.18) (GST class-pi)
B	SW:GSHB_HUMAN	8	pMism:13.37	Glutathione synthetase (EC 6.3.2.3) (glutathione synthase)
A	SW:G3P2_HUMAN	4	pMism:7.38	Glyceraldehyde 3-phosphate dehydrogenase, liver (EC 1.2.1.12)
A	SW:GBLP_HUMAN	7	pMism:10.08	Guanine nucleotide-binding protein beta subunit-like protein 12.3 (p205)
A, B	SW:HS27_HUMAN	7	pMism:13.08	Heat shock 27 kDa protein (HSP 27) (stress-responsive protein 27)
A, B	SW:HS71_HUMAN	8	pMism:13.76	Heat shock 70 kDa protein 1 (HSP70.1) (HSP70-1/HSP70-2)
B	SW:HS76_HUMAN	6	pMism:9.56	Heat shock 70 kDa protein 6 (heat shock 70 kDa protein B′)
A, B	SW:HS7C_HUMAN	9	pMism:13.07	Heat shock cognate 71 kDa protein
B	TR_HUM:AAN65630	6	pMism:6.93	Hepatocellular carcinoma associated protein TB6
A	SW:YHU6_YEAST	6	pMism:7.80	Hypothetical 51.1 kDa protein in DCD1-MRPL6 intergenic region
A	TR_HUM:Q8N1N4	5	pMism:7.81	Hypothetical protein FLJ39100
B	SW:ALC1_HUMAN	6	pMism:13.03	Ig alpha-1 chain C region
B	SW:ALC2_HUMAN	6	pMism:9.44	Ig alpha-2 chain C region
B	SW:GC1_HUMAN	5	pMism:8.28	Ig gamma-1 chain C region
B	SW:KAC_HUMAN	4	pMism:8.20	Ig kappa chain C region
B	SW:MUC_HUMAN	7	pMism:10.04	Ig mu chain C region
B	SW:MUCB_HUMAN	5	pMism:7.55	Ig mu heavy chain disease protein (bot)
B	TR_HUM:AAB3083	5	pMism:8.13	Immunoglobulin A heavy chain allotype 2 (fragment)
B	SW:IGJ_HUMAN	4	pMism:7.78	Immunoglobulin J chain
A	SW:IL1X_HUMAN	4	pMism:8.29	Interleukin-1 receptor antagonist protein precursor (IL-1ra)
A	SW:PLAK_HUMAN	4	pMism:8.04	Junction plakoglobin (desmoplakin III)
A	SW:K1CM_HUMAN	9	pMism:11.83	Keratin, type I cytoskeletal 13 (cytokeratin 13) (K13) (CK 13)
A	SW:K1CN_HUMAN	9	pMism:10.83	Keratin, type I cytoskeletal 14 (cytokeratin 14) (K14) (CK 14)
A	SW:K1CO_HUMAN	6	pMism:8.17	Keratin, type I cytoskeletal 15 (cytokeratin 15) (K15) (CK 15)
A	SW:K1CP_HUMAN	6	pMism:8.48	Keratin, type I cytoskeletal 16 (cytokeratin 16) (K16) (CK 16)
A	SW:K22E_HUMAN	5	pMism:6.69	Keratin, type II cytoskeletal 2 epidermal (cytokeratin 2e)
A	SW:K22O_HUMAN	8	pMism:11.65	Keratin, type II cytoskeletal 2 oral (cytokeratin 2p)
A	SW:K2C3_HUMAN	7	pMism:8.91	Keratin, type II cytoskeletal 3 (cytokeratin 3) (K3) (CK3)
A, B	SW:K2C4_HUMAN	9	pMism:8.81	Keratin, type II cytoskeletal 4 (cytokeratin 4) (K4) (CK4)
A	SW:K2C5_HUMAN	8	pMism:8.38	Keratin, type II cytoskeletal 5 (cytokeratin 5) (K5) (CK 5)
A	SW:K2CA_HUMAN	9	pMism:9.67	Keratin, type II cytoskeletal 6a (cytokeratin 6a) (CK 6a)
A, B	SW:K2CB_HUMAN	9	pMism:9.16	Keratin, type II cytoskeletal 6b (cytokeratin 6b) (CK 6b)
A	SW:K2CD_HUMAN	8	pMism:10.74	Keratin, type II cytoskeletal 6d (cytokeratin 6d) (CK 6d)
A, B	SW:K2CE_HUMAN	9	pMism:9.76	Keratin, type II cytoskeletal 6e (cytokeratin 6e) (CK 6e)
A	SW:K2CF_HUMAN	6	pMism:7.70	Keratin, type II cytoskeletal 6f (cytokeratin 6f) (CK 6f)
A, B	SW:TRFL_HUMAN	9	pMism:9.42	Lactotransferrin precursor (lactoferrin)
B	SW:ILEU_HUMAN	6	pMism:9.37	Leukocyte elastase inhibitor (LEI) (monocyte/neutrophil elastase inhibitor)
A, B	SW:LKHA_HUMAN	9	pMism:14.08	Leukotriene A4 hydrolase (EC 3.3.2.6) (LTA-4 hydrolase)
B	SW:PLSL_HUMAN	7	pMism:8.10	L-plastin (lymphocyte cytosolic protein 1) (LCP-1)
A	SW:CAPG_HUMAN	4	pMism:7.59	Macrophage capping protein (actin-regulatory protein CAP-G)
A	SW:MDHM_HUMAN	7	pMism:8.25	Malate dehydrogenase, mitochondrial precursor (EC 1.1.1.37)
A	SW:MASP_HUMAN	9	pMism:12.66	Maspin precursor (protease inhibitor 5)
A	SW:PRN3_HUMAN	4	pMism:11.33	Myeloblastin precursor (EC 3.4.21.76) (leukocyte proteinase 3)
B	SW:MY10_HUMAN	5	pMism:7.16	Myosin X
A	SW:NAGK_HUMAN	7	pMism:12.42	N-acetylglucosamine kinase (EC 2.7.1.59)
B	SW:CYPH_HUMAN	4	pMism:7.34	Peptidyl-prolyl cis-trans isomerase a (EC 5.2.1.8) (PPIase)
A	SW:PDX1_HUMAN	8	pMism:13.72	Peroxiredoxin 1 (thioredoxin peroxidase 2)
A	SW:PDX2_HUMAN	4	pMism:11.27	Peroxiredoxin 2 (EC 1.11.1.) (thioredoxin peroxidase 1)
A	SW:PDX5_HUMAN	5	pMism:10.87	Peroxiredoxin 5, mitochondrial precursor (Prx-V)
A, B	SW:PMG1_HUMAN	7	pMism:11.60	Phosphoglycerate mutase 1 (EC 5.4.2.1) (EC 5.4.2.4)
B	SW:PIGR_HUMAN	9	pMism:14.20	Polymeric-immunoglobulin receptor precursor (poly-Ig receptor)
A	SW:PBEF_HUMAN	5	pMism:8.46	Pre-B cell enhancing factor precursor
A, B	SW:PRO1_HUMAN	6	pMism:11.37	Profilin I
A, B	SW:PIP_HUMAN	6	pMism:15.52	Prolactin-inducible protein precursor (secretory actin-binding protein)
B	SW:PDI1_HUMAN	6	pMism:7.53	Protein-arginine deiminase type I (EC 3.5.3.15)
A	SW:TGM3_HUMAN	8	pMism:10.61	Protein-glutamine glutamyltransferase E precursor (EC 2.3.2.13)
B	SW:PNPH_HUMAN	5	pMism:8.02	Purine nucleoside phosphorylase (EC 2.4.2.1)
A, B	SW:PDXK_HUMAN	5	pMism:8.21	Pyridoxine kinase (EC 2.7.1.35)
A	SW:KPY1_HUMAN	7	pMism:8.89	Pyruvate kinase, M1 isozyme (EC 2.7.1.40)
B	SW:GDIB_HUMAN	8	pMism:9.67	Rab GDP dissociation inhibitor beta (Rab GDI beta)
A	SW:IQG1_HUMAN	8	pMism:8.74	Ras GTPase-activating-like protein IQGAP1 (p195)
B	TR_HUM:CAC24982	5	pMism:9.62	Sequence 26 from patent wo0100806 precursor
A, B	SW:TRFE_HUMAN	9	pMism:9.41	Serotransferrin precursor (transferrin) (siderophilin)
A, B	SW:ALBU_HUMAN	9	pMism:14.83	Serum albumin precursor
A, B	SW:SCC1_HUMAN	7	pMism:8.98	Squamous cell carcinoma antigen 1 (SCCA 1)
A	SW:GR75_HUMAN	7	pMism:12.04	Stress-70 protein, mitochondrial precursor (75 kDa glucose-regulated protein)
B	SW:SYNP_HUMAN	4	pMism:7.15	Synphilin 1 (alpha-synuclein interacting protein)
A	SW:TKT_HUMAN	5	pMism:8.13	Transketolase (EC 2.2.1.1) (TK)
A	SW:TPIS_HUMAN	7	pMism:11.79	Triosephosphate isomerase (EC 5.3.1.1) (TIM)
A	SW:TBA1_HUMAN	8	pMism:16.58	Tubulin alpha-1 chain (alpha-tubulin 1)
A	SW:TBA4_HUMAN	7	pMism:12.89	Tubulin alpha-4 chain
A	SW:KCY_HUMAN	4	pMism:11.58	UMP-CMP kinase (EC 2.7.4.14) (cytidylate kinase)
A	SW:POR1_HUMAN	5	pMism:10.28	Voltage-dependent anion-selective channel protein 1 (VDAC 1)
A	SW:POR2_HUMAN	5	pMism:11.75	Voltage-dependent anion-selective channel protein 2 (VDAC 2)
A	SW:WDR1_HUMAN	5	pMism:8.27	WD-repeat protein 1 (actin interacting protein 1)
B	SW:ZA2G_HUMAN	9	pMism:13.3	Zink-alpha-glycoprotein precursor

A = epithelial cell debris, B = supernatant.

**Table 2:** **List 2: identified proteins in human saliva**

SW name	cMS control	cMS HNSCC	Full protein name	Acc. Nr.
sw:ENOA_HUMAN	0	33	Alpha enolase (EC 4.2.1.11) (2-phospho-D-glycerate hydro-lyase) (non-neural enolase) (NNE) (enolase 1) (phosphopyruvate hydratase)	P06733 Q16704 Q9UM55
sw:K1CQ_HUMAN	0	17	Keratin, type I cytoskeletal 17 (cytokeratin 17) (K17) (CK 17) (39.1)	Q04695
sw:G3P2_HUMAN	0	12	Glyceraldehyde 3-phosphate dehydrogenase, liver (EC 1.2.1.12) (GAPDH)	P04406
sw:FSC1_HUMAN	0	11	Fascin (singed-like protein) (55 kDa actin bundling protein) (p55)	Q16658 Q96IC5 Q9BRF1
sw:HBA_HUMAN	0	11	Hemoglobin alpha chain	P01922
sw:MASP_HUMAN	0	11	Maspin precursor (protease inhibitor 5)	P36952
sw:NDR1_HUMAN	0	11	NDRG1 protein (N-myc downstream-regulated gene 1 protein) (differentiation-related gene 1 protein) (DRG1) (reducing agents and tunicamycin-responsive protein) (RTP) (nickel-specific induction protein CAP43) (RIT42)	Q92597 O15207 Q9NYR6 Q9UK29
sw:PGK1_HUMAN	0	11	Phosphoglycerate kinase 1 (EC 2.7.2.3) (primer recognition protein 2) (PRP 2)	P00558
sw:PLSL_HUMAN	0	10	L-plastin (lymphocyte cytosolic protein 1) (LCP-1) (LC64P)	P13796
sw:SCC1_HUMAN	0	10	Squamous cell carcinoma antigen 1 (SCCA-1) (protein T4-A)	P29508 Q96J21
sw:TYPH_HUMAN	0	10	Thymidine phosphorylase precursor (EC 2.4.2.4) (TdRPase) (TP) (platelet-derived endothelial cell growth factor) (PD-ECGF) (gliostatin)	P19971 Q13390 Q8WVB7
sw:CATA_HUMAN	0	8	Ccatalase (EC 1.11.1.6)	P04040
humangp:HCHR12-007545	0	6	tr:q7z793: keratin 6 L was used to identify this gene current sequence which covers 100.0% of the above current sequence which is 100.0% identical to the above sequence	HCHR12-007545
sw:TBB5_HUMAN	0	6	Tubulin beta-5 chain	P05218 Q8WUC1 Q9CY33
sw:TBBX_HUMAN	0	6	Tubulin beta-5 chain (tubulin 5 beta)	P04350
sw:TKT_HUMAN	0	6	Transketolase (EC 2.2.1.1) (TK)	P29401
sw:TAL1_HUMAN	0	5	Transaldolase (EC 2.2.1.2)	P37837 O00751
sw:VINC_HUMAN	0	5	Vinculin (metavinculin)	P18206 Q16450
sw:CAH2_HUMAN	0	4	Carbonic anhydrase II (EC 4.2.1.1) (carbonate dehydratase II) (CA-II) (carbonic anhydrase C)	P00918 Q96ET9
sw:CERU_HUMAN	0	4	Ceruloplasmin precursor (EC 1.16.3.1) (ferroxidase)	P00450 Q14063
sw:GTO1_HUMAN	0	4	Glutathione transferase omega 1 (EC 2.5.1.18) (GSTO 1-1)	P78417
sw:ITH4_HUMAN	0	4	Inter-alpha-trypsin inhibitor heavy chain H4 precursor (ITI heavy chain H4) (inter-alpha-inhibitor heavy chain 4) (inter-alpha-trypsin inhibitor family heavy chain-related protein) (IHRP) (plasma kallikrein-sensitive glycoprotein 120) (PK-120) (GP120) (Pro1851) [contains: GP57]	Q14624 Q15135 Q9P190 Q9UQ54
sw:OTB1_HUMAN	0	4	Ubiquitin thiolesterase protein OTUB1 (EC 3.4.-.-) (otubain 1) (OTU domain-containing ubiquitin aldehyde-binding protein 1) (ubiquitin-specific processing protease OTUB1) (deubiquitinating enzyme OTUB1) (HSPC263)	Q96FW1 Q96II3 Q9NXQ4 Q9P0B8
sw:PLST_HUMAN	0	4	T-plastin	P13797
sw:SAHH_HUMAN	0	4	Adenosylhomocysteinase (EC 3.3.1.1) (s-adenosyl-l-homocysteine hydrolase) (AdoHcyase)	P23526 Q96A36

Cofilin, C-reactive protein precursor, creatine kinase M-chain, fatty acid binding protein, keratin type II, myosin light chain 2 and 3, nucleoside diphosphate kinase A, phosphoglycerate mutase 1, plakoglobulin and retinoic acid binding protein II were not previously described to be differentially expressed in the OSCC proteome of saliva. Figures 1 and 2 show examples of 2DE-PAGES of saliva samples from OSCC patients. The identified proteins that were expressed at altered levels have various functions. They are involved in cellular transport and chaperoning, like the heat shock protein (27 kDa) and the heat shock-like protein p20 (20 kDa), in regulatory functions, like creatine kinase, pyruvate kinase M1 and nucleoside diphosphate kinease A, or in the glycolic pathway, like phosphoglycerale mutase and argininosuccinate synthase. Cytoskeletal proteins of the tropomyosin family were also identified (see Table 1). Some of the proteins are highly tissue specific, like galectin-7, which has been shown to be restricted to the stratified epithelium and is involved in the induction of pro-apoptotic functions and the cell-cell or cell-matrix adhesion.

### Results from the OSCC tissue proteome study

**Figure 3:**
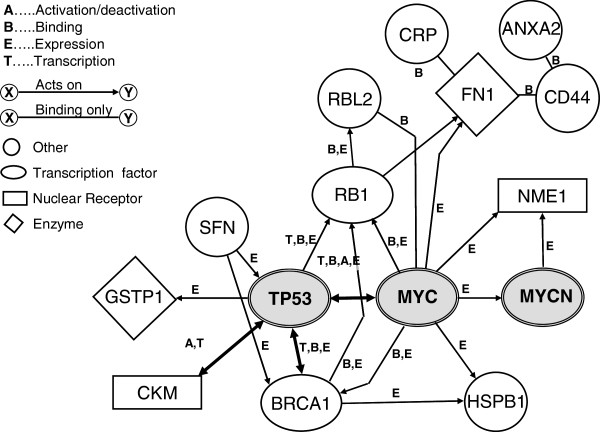
**Schematic result of proteomics study analysed by pathway analysis.** The diagram was constructed with the use of the Ingenuity Pathway Analysis software as described in the ‘Methods’ and ‘Results’ sections. ANXA2, annexin A2; BRCA1, breast cancer 1; CKM, creatinine kinase M-chain; CRP, c-reactive protein; FN1, fibronectin 1; GSTP1, glutathione S-transferase pi; HSPB1, heat shock protein 27; NME1, nucleoside diphosphate kinase A; RB1, retinoblastoma; RBL2, retinoblastoma-like protein 2; SFN, 14-3-3sigma, stratifin.

In our previous studies [5], OSCC tumour and control tissue from different patients were analysed by proteomic analysis. Furthermore, the results were compared to the list of the conducted saliva proteome study. From the tumour tissues, 350 proteins were identified, of which 16 proteins were upregulated in tumour tissue while 4 were downregulated in the tumours [5]. Using pathway analysis, most of the proteins overexpressed in tumours could be mapped to the p53, c-Myc and N-Myc pathways and showed a specific induction to the core proteins in OSCC tumour samples (see Figure 3). While some of the proteins that were identified as potential biomarkers for OSCC in our proteomics/pathway study were shown to be associated to OSCC before, e.g. *squamous cell carcinoma antigen 1* (SCCA-1) and 14-3-3sigma (stratifin), others have not yet been identified. Extending the pathway analysis, several other biomarker candidates were identified that could be used for diagnosis of OSCC in saliva samples.

### Results from saliva proteome study

**Figure 4:**
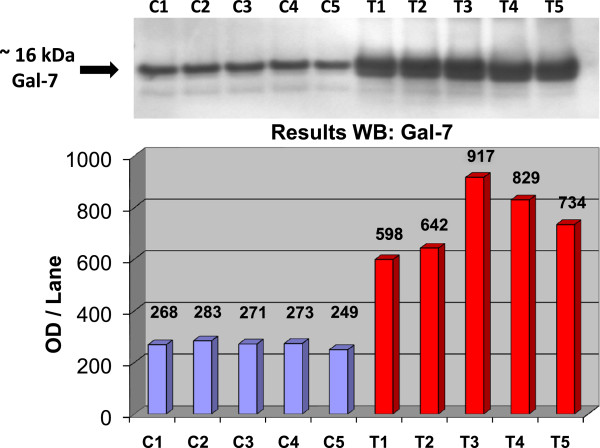
**Western blot on five saliva samples derived from OSCC and healthy controls was used for validation of galectin-7.** B-actin was normalised for WB content, and equal amounts were separated on a SDS-PAGE gel and detected by galectin-7 specific antibodies.

Fractions of supernatant and cell proteins were prepared from saliva samples of each patient as described in the ‘Methods’ section. The whole saliva was used for fractioning taking into consideration that differences in cell distribution might exist. Additionally, this approach diminishes artifacts that could have been introduced during the preparation of saliva samples. The enriched supernatant and cell debris proteins in the corresponding sub-fractions were assessed by 2-D gels. Thus, α-enolase and pyruvate kinase were mainly present in the supernatant fraction, whereas the peroxisomal protein catalase, which was co-isolated with the cell debris proteins, was present in the insoluble fraction.Preliminary results of our study show, e.g. that galectin-7 can be efficiently detected in the saliva of healthy and tumour bearing patients and is strongly overrepresented in tumour saliva samples (see Figure 4). Other OSCC biomarker candidates like stratifin, SCCA-1 and kallikrein-7 will be analysed as well.

For the initial validation of biomarkers and antibodies, quantitative Western blots will be used. After the suitability of the biomarker has been confirmed highly sensitive, ELISA assays or protein arrays will be developed using only highly specific antibodies.

### Discussion

In the present study, the protein levels in saliva from OSCC and control patients were quantified with the goal to detect differences in the protein concentrations that could serve as markers for the disease. Diagnosis of OSCC can be difficult in certain clinical situations, even with histological examination of the lesion. Therefore, the use of saliva or serological markers may allow earlier tumour detection and timely intervention. To be useful as a screening or stratification marker, a protein should be expressed in minimal amounts. A differential expression of at least 1.5-fold was chosen in the present study since this difference enables a reproducible detection by current technologies. The question exists to what percentage a protein should be overexpressed in a tumour to be a reliable marker candidate as most of the differentially expressed proteins identified in saliva have basal expression levels in healthy patients as well. We found 25 proteins with altered expression levels in saliva from OSCC patients (see Table 2), some of which, like phosphoglycerate mutase, glutathione S-transferase, retinoic acid binding protein II, cofilin, galectin-7 and C-reactive protein, have been already described in cancer cell lines [37, 38] but not in the saliva of OSCC patients. Altered expression of certain proteins that were observed in the present study has been previously described by other groups that used the proteomic approach as well [39].

**Figure 5:**
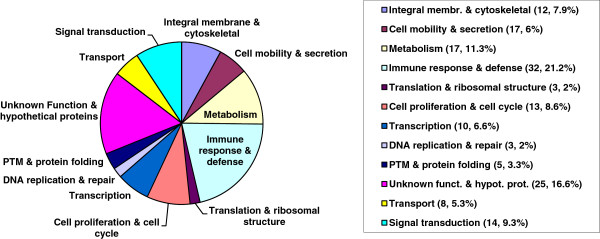
**Important molecular functions and implications on cell shape and size.**

We found that keratin type II is overexpressed not only in OSCC tissues but also in the saliva, which is in agreement with literature data [40]. Thus, the corresponding gene product was found to be upregulated in different squamous cell carcinomas and in its hyperproliferative states. Analysis of 141 epidermoid cancers of head and neck showed that 96% of tumours were positive for the keratin type II protein [41]. Its overexpression in OSCC cells may have important molecular functions as structural constituents of the cytoskeleton as well as implications on cell shape and cell size (see Figure 5).

#### Squamous cell carcinoma antigen 1

SCCA-1 may act as a protease inhibitor to modulate the host immune response against tumour cells. It is exclusively expressed in the cytosol of epithelial cancers and also secreted in plasma by cancerous cells at a low level. It is thought to be involved in the regulation of proliferation of carcinoma cells. SCCA-1 may be useful in specific immunotherapy for cancer patients and may serve as a paradigmatic tool for the diagnosis and treatment of patients with OSCC.

#### Creatine kinase M-chain

In OSCC patients, higher levels of creatine kinase M-chain (CKM) were found too. CKM is a cytoplasmic enzyme and member of the ATP guanido phosphotransferase protein family, which reversibly catalyses the transfer of phosphate between ATP and various phosphogens such as creatine phosphate. CKM is involved in enzyme homeostasis and is an important serum marker for myocardial infarction. It plays an important role in morphology, aggregation and permeability of cells. The levels of glutathione S-transferase were increased in the saliva and tumour as well. Glutathione S-transferases are a family of enzymes that play important roles in detoxification by catalysing the conjugation of many hydrophobic and electrophilic compounds with reduced glutathione. Glutathione S-transferase pi gene is a polymorphic gene, encoding functionally different proteins that are thought to be involved in xenobiotic metabolism and play a role in susceptibility to cancer and other diseases.

#### Retinoic acid-binding protein II

Retinoic acid-binding protein II is a specific carrier protein belonging to a group of analogues that have a profound effect on the growth and differentiation of normal, pre-malignant and malignant epithelial cells *in vitro* as well as *in vivo*. They have the ability to suppress carcinogenesis in various epithelial tissues, such as oral cavity, skin, lung, bladder, prostate and mammary glands. Experimental data in humans have demonstrated that oral administration of different isoforms of retinoids can prevent pre-cancerous (squamous metaplastic) oral and bronchial lesions from progressing to invasive OSCC and can suppress oral second primary carcinoma in patients with lung SCC or HNSCC. We found increased levels of retinoic acid-binding protein in OSCC tissue (Table 1). *In vitro* studies showed that retinoic acid-binding protein suppresses the proliferation of HNSCC cell lines and inhibits the formation of SCC colonies [42]. The mechanism of retinoic acid-mediated regulation of human skin growth and differentiation remains unknown. It is possible that the observed effect is the consequence of the fact that retinoic acid enhances the growth and modulates the differentiation of mucosal epithelial cells. It is also postulated that the retinoic acid-binding gene is transcriptionally regulated by a newly synthesised regulatory protein which has the ability to modulate the transcriptional regulatory activity of a set of nuclear retinoic acid receptors [42].

#### Nucleoside diphosphate kinase A

NME1, which was overexpressed in OSCC, plays an important role in cell movement, invasiveness, disease stage and tumour genesis. Reduced transcript levels of NME1 were previously identified by genome analysis in highly metastatic cells. The NME1 gene encodes for the A isoform of nucleoside disphophate kinase (NDK). Mutations in NME1 have been identified in aggressive neuroblastomas. NME1 is regulated by MYC, MYCN and tumour protein p53.

#### Galectin-7

Galectin-7 showed increased levels in OSCC tumours as well as in saliva samples (see Figure 4). Galectin-7 is a carbohydrate-binding protein, has the ability to bind to complementary molecules in the extracellular matrix or on the surface of other cells, is involved in cell-cell and cell-matrix interactions and is necessary for normal cell growth control. Expression of galectin-7 is positively altered in certain tumours that exhibit an aggressive phenotype. Its expression pattern appears to be associated with the degree of squamous differentiation, suggesting a potential utilisation of galectin-7 as a biological and differentiation marker in OSCC.

#### The heat shock proteins, HSP27, HSP60, HSP71, HSP90

As well as the calcium-binding proteins, calreticulin and calnexin, were also previously detected in other cancer cells [35]. However, the stress proteins are present in large amounts in both normal epithelial and tumour cells, making their potential for use as clinical markers negligible. It has been shown that heat shock proteins (HSPs) also participate in essential physiological processes, such as regulation of cell cycle, differentiation, programmed cell death and tumourigenesis. Small heat shock proteins include HSP60, HSP27, HSP20 and alpha B-crystallin. HSP20 is transiently expressed during cell division to differentiate transition, and this phenomenon prevents differentiating cells from undergoing apoptosis. HSP20 also protects cells from apoptosis induced by different stimuli or agents, particularly anti-cancer drugs. Interestingly, tumour cells usually express high levels of HSP20 and anti-cancer drugs, like cisplatin, which triggers the accumulation of HSP20. HSP27 and HSP20 are independently modulated in response to stress [43]. The overexpression of HSP27 and the downregulation of HSP20 in OSCC observed in this study are consistent with the results from studies of other cancers [44].

The proteins underexpressed in OSCC saliva include annexin I, heat shock 20 kDa-like protein p20, plakoglobin and myosin light chains 1, 2 and 3. These proteins were present in significant levels in normal epithelial tissues, rendering them clinically irrelevant. Annexin I (ANXA1) belongs to a family of Ca^2+^-dependent, phospholipid-binding proteins that have been implicated in a broad range of molecular and cellular processes, including modulation and inhibition of phospholipase A2 and kinase activity, in signal transduction, the maintenance of cytoskeleton and extracellular matrix integrity, tissue growth and differentiation, inflammation and blood coagulation. ANXA1 plays a major regulatory role in cell growth regulation and differentiation, neutrophil migration, central nervous system response to cytokines, neuroendocrine secretion and mediation of apoptosis [39]. It is normally expressed at high levels in a wide range of organs and tissues, is specifically implicated in epithelial differentiation and growth regulation and is markedly downregulated in certain other cancers [45], including esophageal squamous cell carcinomas [46]. Using immunoblots, Bouden and Krieg [47] found downregulation of annexin I in head and neck cancer, which agrees with our results. Contrary to these results, Paweletz et al. [48] used a proteomics approach and observed increased levels for annexin I in buccal squamous cell carcinoma [48]. Since phospholipase A2 is required for biosynthesis of the potent mediators of inflammation, prostageomdins and leukotriens, ANXA1 may have potential anti-inflammatory activity.

Carcinogenesis of squamous cells involves alterations of the adhesive properties of cells to each other as well as to the basement membrane. In epidermal keratinocytes, the main cell-cell adhesion systems are adherens junctions and desmosomes. Plakoglobin is one of the desmosome components and was reported to be expressed in various skin carcinomas such as basal cell carcinoma (BCC), SCC, extra mammary Paget’s disease and Bowen’s disease [49]. In normal human skin, plakoglobin is strongly expressed in the intercellular space of the epidermis except of the basal cell layer. We observed that expression of plakoglobin in OSCC was reduced or was absent in tumour cells. Decreased expression of plakoglobin in skin carcinomas is associated with the invasive and metastatic ability of tumour cells [50].

## Expert recommendations

Protein analysis of saliva as a clinical application offers an attractive, simple and rapid diagnosis tool for the short- and long-term monitoring of pathological disorders and drug therapy. The collection of saliva, either in the pure or in the whole form, is a relatively easy and non-invasive procedure that is not harmful to the patients and causes no complications at all. The present study discusses the identification of tumour-related proteins in saliva by proteome analysis, which can be used for detection and identification of possible marker proteins, specific for OSCC in the head and neck. In the course of our study, we validated Gal-7 as a potential screening by Western blot analysis. The results showed a specificity of around 90% and a sensitivity of 80% (*n* = 10), meaning that Gal-7 is a good screening marker for diagnosis of OSCC in saliva.

### Outlook

Most studies published so far have analysed the OSCC proteome or genome in tissue biopsies. Though the information gained from such studies is important for understanding the mechanisms of carcinogenesis and can lead to the identification of biomarkers or therapeutic targets. However, biopsies are not suitable for screening purposes. For early diagnosis and screening of risk populations, markers that can predict the development of malignancies at an early stage or even in a precancerous stage would be invaluable. Therefore, biomarkers in blood and, especially in the case of OSCC, saliva are now a focus of research. Screening of saliva as a clinical application offers an attractive, simple and rapid diagnosis tool for the short- and long-term monitoring of pathological disorders and drug therapy. To collect saliva, either in the pure or in the whole form is a relatively easy and a non-invasive procedure, and it is not harmful to the patients and has no complications. Further validation of saliva markers are still in process and will follow recommendations of the “EPMA White Paper” [50]. Finally, the authors consider to create a topic-relevant multidisciplinary projects responding the needs of the European population (innovative screening programmes, monitoring of chronic diseases, patient self-management, etc.) in the course of the scientific calls of “Horizon 2020” [51].
